# Change in Antinuclear Antibodies After Lung Transplantation in Patients with Systemic Sclerosis

**DOI:** 10.3390/jcm14248673

**Published:** 2025-12-07

**Authors:** Víctor Barreales-Rodríguez, Alfredo Guillen-Del-Castillo, Cristina Berastegui, Manuel López-Meseguer, Víctor Monforte, Berta Saez-Gimenez, Ana Villar, Iñigo Ojanguren, Claudia Codina-Clavaguera, Alejandra Fernández-Luque, María Teresa Sanz-Martínez, Laura Viñas-Giménez, Janire Perurena-Prieto, Laura Triginer-Gil, Luis Alcalá-González, Carlos Bravo, Carmen Pilar Simeón Aznar

**Affiliations:** 1 Internal Medicine Service, University Health Care Complex of León, 24008 Leon, Spain; victorbarreales@gmail.com; 2 Autoimmune Diseases Unit, Internal Medicine Department, Vall d’Hebron University Hospital, 08035 Barcelona, Spain; 3 Lung Transplant and Pulmonary Vascular Pathology Unit, Pneumology Department, Vall d’Hebron University Hospital, 08035 Barcelona, Spainmanuel.lopez@vallhebron.cat (M.L.-M.); ana.villar@vallhebron.cat (A.V.);; 4 Centro de Investigación Biomédica en Red, Enfermedades Respiratorias, 28003 Madrid, Spain; 5 ERN-LUNG (European Reference Network on Rare Respiratory Diseases), 08035 Barcelona, Spain; 6 Interstitial Lung Disease Unit, Department of Pulmonology, Vall d’Hebron University Hospital, 08035 Barcelona, Spain; 7 Department of Immunology, Vall d’Hebron University Hospital, 08035 Barcelona, Spain; 8 Digestive System Research Unit, Department of Digestive Diseases, Vall d’Hebron University Hospital, 08035 Barcelona, Spain

**Keywords:** systemic sclerosis, lung transplantation, autoantibodies, antinuclear antibodies

## Abstract

**Objectives:** Lung transplantation (LT) is a rescue therapy for end-stage pulmonary diseases, including systemic autoimmune diseases. The aim of this study was to analyse the evolution of patients with systemic sclerosis (SSc) who, after undergoing LT, become negative for antinuclear antibodies (ANA) and to assess whether they have different clinical and prognostic characteristics than patients who do not become negative. **Material and Methods:** A retrospective, descriptive analysis was performed over a cohort of patients with a diagnosis of SSc, who underwent unilateral or bilateral LT between 2006 and 2021 at the Vall d’Hebron University Hospital. Clinical and analytical data were obtained from these patients by reviewing their electronic medical records. Two groups of patients were compared: those who tested negative for ANA after LT and those who did not. Statistical analysis was performed with SPSS Statistics 20.0. **Results:** Eighteen patients were included. The most frequent indication for LT was interstitial lung disease (ILD) combined with pulmonary hypertension (PH), in 13 (72%) patients. All had ANA before the LT (n = 18), and regarding specific SSc autoantibodies, anti-topoisomerase I was presented in 44% (n = 8), anti-U11/U12RNP in 17% (n = 3), anti-RNA Polymerase III in 11.1% (n = 2), anti-Ro52 in 11% (n = 2) and anti-centromere in 6% of individuals (n = 1). 39% (n = 7) of the patients had negative post-LT ANA, 44% (n = 8) had declining titres, and 17% (n = 3) had stable ANA titres. Titres did not increase in any case after LT. Those patients who became ANA-negative after LT were those who had significantly lower titres before LT. No statistically significant differences between groups were found related to pre-LT clinical characteristics, immunosuppressive regimen applied after LT, or in post-LT outcomes. A non-significant trend towards better survival was observed in patients who became ANA negative, with a cumulative survival at 5 years of 85.7% compared to 72.7% among those who remained ANA-positive. **Conclusions:** Most patients with SSc clear ANA or reduce their levels after LT. A trend towards better survival was observed in this group, compared to the group of transplanted patients who remained positive.

## 1. Introduction

Systemic sclerosis (SSc) is a multisystem autoimmune disease characterised by progressive skin fibrosis and other organs, vasculopathy, and immune dysregulation in other organs. It is recognised as one of the systemic autoimmune diseases with the highest mortality rates; nevertheless, advances in therapeutic strategies have contributed to a notable improvement in prognosis in recent years [1,2]. The prevalence ranges between 38 and 341 cases per million [3]. The 2013 ACR/EULAR Classification Criteria [4] are currently defined, with high sensitivity and specificity [5]. Based on the extent of cutaneous involvement, SSc is subclassified into limited cutaneous SSc (lcSSc), diffuse cutaneous SSc (dcSSc), and sine scleroderma SSc.

Visceral manifestations are frequent and play a key role in the quality of life [6] and prognosis. Currently, pulmonary involvement is the leading cause of disease-related mortality [7,8]. It most commonly presents as interstitial lung disease (ILD) or pulmonary arterial hypertension (PAH). Although recent therapeutic advances have led to improved clinical outcomes [9,10,11], disease progression may still occur, making lung transplantation (LT) a necessary last resort [12,13].

Approximately 95% of patients with SSc exhibit antinuclear antibodies (ANA) [14]. The most prevalent SSc-specific autoantibodies include anticentromere antibodies (ACAs), anti-topoisomerase-I (ATA I), and anti-RNA polymerase-III (anti-RNApol III) [15]. These autoantibodies are associated with distinct clinical phenotypes and patterns of organ involvement [16,17] and are independent prognostic factors. For example, patients with dcSSc and ATA I positivity tend to exhibit worse survival and a higher prevalence of ILD compared to those with lcSSc and ATA I positivity [18]. Salazar analysed 3249 patients with SSc, identifying a 6.2% prevalence of ANA negativity. ANA-negative individuals displayed a milder clinical phenotype, with a lower incidence of vasculopathic features (e.g., PAH and digital ulcers), although overall survival did not differ significantly [19]. Similarly, Hudson analysed 800 patients with SSc, 5% of whom were ANA-negative, and reported a more favourable clinical course and prognosis [20]. Case reports have described seroconversion or a decline in ANA titres following immunosuppressive therapy with methotrexate [21] and rituximab [22,23]. Furthermore, cases have been reported of cutaneous clinical improvement after ANA disappearance following bilateral LT [24]. However, to date, no studies have demonstrated a potential correlation between the ANA clearance and survival outcomes in SSc patients undergoing LT.

The present study aims to characterise the clinical and demographic characteristics of SSc patients who become ANA-negative following LT, and to determine whether such disappearance is associated with distinct clinical features and prognostic implications compared to patients who remain ANA-positive.

## 2. Materials and Methods

### 2.1. Ethics

The study protocol was approved by the Ethics Committee for Clinical Research (PG(AG)4/2015). All participants provided written informed consent to participate in accordance with the principles outlined in the Declaration of Helsinki.

### 2.2. Study Design and Population

A retrospective observational descriptive study was conducted on a cohort of 18 patients diagnosed with SSc and pulmonary involvement who underwent either single or bilateral LT between January 2006 and December 2021 at Vall d’Hebron University Hospital (Barcelona, Spain). This cohort accounted for 1.6% of the 1085 patients who underwent LT at this centre during the same period. Clinical management was coordinated between the Lung Transplantation and Pulmonary Vascular Pathology Unit of the Pneumology Department and the Systemic Autoimmune Diseases Unit of the Internal Medicine Department. All patients fulfilled the 2013 ACR/EULAR classification criteria for SSc [5]. Patients were subclassified according to the LeRoy and Medsger subtypes [25]: limited cutaneous SSc (lcSSc), defined as cutaneous involvement distal to the elbows and knees; diffuse cutaneous SSc (dcSSc), defined as cutaneous involvement proximal and distal to the elbows and knees; and *sine* scleroderma SSc, defined as visceral involvement without cutaneous sclerosis. Pulmonary hypertension (PH) was defined as a mean pulmonary artery pressure ≥ 20 mmHg as measured by right catheterisation, with a pulmonary arterial wedge pressure ≤ 15 mmHg, and with pulmonary vascular resistance > 2 Woods units (WUs) in accordance with the 2022 ESC/ERS Guidelines for the diagnosis and treatment of pulmonary hypertension [26]. PH group 1 or 3 was determined following the guidelines definitions [26]. ILD was diagnosed via high-resolution computed tomography (HRCT). Pulmonary function data, including the forced vital capacity (FVC) percentage and the carbon monoxide diffusion capacity (DLCO) percentage prior to lung transplantation, were collected.

Clinical and laboratory data were collected from the hospital’s electronic medical records at the first visit to Vall d’Hebron University Hospital, during the perioperative period, and throughout the post-transplant follow-up period.

Patients were divided into two groups based on their ANA serostatus after LT: those who became ANA-negative and those who remained ANA-positive. The clinical and epidemiological characteristics of both groups (including age and sex) were compared prior to LT, as were the types of pulmonary involvement and the presence of other visceral manifestations such as Raynaud’s phenomenon (RP) [27] (episodic vasospastic attacks of the fingers or toes triggered by cold or stress, leading to pallor or cyanosis), digital ulcers [28] (painful ischaemic lesions on the fingertips or periungual areas due to microvascular compromise in SSc), gastrointestinal involvement [29,30] (oesophageal dysmotility confirmed by oesophageal manometry or scintigraphy; gastric involvement when hypomotility was confirmed by gastric emptying scintigraphy, or when gastric antral vascular ectasia was observed by upper-endoscopy; or intestinal involvement when either a motility alteration by scintigraphy or intestinal manometry was confirmed, malabsorption confirmed by breath test, or intestinal pseudo-obstruction confirmed by an abdominal scan); musculoskeletal involvement (arthritis; myositis, which is defined as muscle weakness, elevation of creatine kinase levels and a myopathic pattern in the electromyogram and signs of inflammation in a muscle biopsy; tendon contractures or friction rubs; calcinosis) [31], cardiac involvement [32] (pericardial involvement, such as pericarditis or pericardial effusion, left ventricular diastolic dysfunction or left ventricular ejection fraction lower than 50% determined by echocardiography), and scleroderma renal crisis [33] (defined as sudden onset of severe hypertension and acute kidney injury). The presence of a late capillaroscopy pattern was evaluated using nailfold videocapillaroscopy (Optilia Instruments AB, Sollentuna, Sweden). Data on the immunosuppressive regimen administered prior to LT were collected, including corticosteroids and the median prednisone daily dose equivalent, mycophenolate acid and the median daily dose, intravenous cyclophosphamide and median accumulated dose, and the use of rituximab or azathioprine.

The type of surgical procedure, post-transplant immunosuppressive treatment, and transplant-related complications, including acute rejection episodes, infectious events, and chronic lung allograft dysfunction (CLAD), were also evaluated. CLAD was defined as a progressive and irreversible decline in graft function, characterised by persistent airflow limitation or restrictive allograft physiology more than three months after transplantation.

### 2.3. Immunological Assessment

All immunological analyses were performed by experienced personnel in the central immunology laboratory of Vall d’Hebron University Hospital following standardised protocols. ANA were determined at the time of SSc diagnosis by indirect immunofluorescence (IIF) using HEP-2 cells as substrate (INOVA, San Diego, CA, USA). Titres were determined by two-fold serial endpoint dilution, and ANA positivity was defined as a titre equal to or greater than 1:80 [34]. The fluorescence pattern (speckled, nucleolar, centromeric, homogeneous, or mixed) was recorded for each patient. Titres were subsequently monitored following LT every 6 months. Specific autoantibodies were identified using the EURO-LINE Systemic Sclerosis Profile (Euroimmun, Lübeck, Germany), which detects anticentromere proteins A and B, anti-topoisomerase I, anti-RNA-polymerase III, anti-PM/Scl proteins PM/Scl100 and PM-Scl75, anti-U3-RNP, anti-Th/To, and anti-Ku antibodies. Anti-U11/U12RNP antibodies were tested using a novel particle-based multi-analyte technology against RNPC-3 (PMAT, Inova Diagnostics, San Diego, CA, USA), and this was confirmed by RNA immunoprecipitation (IP) [35]. Antinuclear valosin-containing protein-like (NVL) antibodies were determined using a non-radioactive protein IP protocol and Western blot assay [36,37].

### 2.4. Statistical Analysis

Data were collected and managed in a secure electronic database (Microsoft Access). Categorical variables were analysed using Fisher’s exact test or chi-square test. Continuous variables were analysed by the Mann–Whitney U test. Survival analysis was performed using the Kaplan–Meier method and Cox regression analysis. All statistical analyses were conducted using SPSS Statistics v20.0 (SPSS Inc., Chicago, IL, USA). Statistical significance was defined as a *p*-value < 0.05.

## 3. Results

### 3.1. Clinical–Epidemiological Characteristics

During the study period, a total of 25 patients with SSc underwent LT. Seven patients were excluded due to transfer of care to other institutions, loss of follow-up, insufficient clinical data beyond the first post-LT year, or the absence of ANA test results, resulting in a final cohort of 18 patients included in the analysis. The participants were predominantly female, accounting for 67% (n = 12), and the mean age of the participants was 54.4 years, the age at SSc onset was 42.4 years, and the age at SSc diagnosis was 43.2 years. 50% and 44% were classified as lcSSc and dcSSc, respectively. Of these, 88% (n = 16) had ILD, 72% (n = 13) had associated PH (PH group 3), and only two patients (11%) had PAH (PH group 1). Bilateral LT was performed in 83% (n = 15) of cases. In the study of respiratory function tests prior to lung transplantation, the median FVC percentage was 50.7%, and the median DLCO was 20%.

Regarding baseline SSc manifestations, most patients presented with RP (94%) and digital ulcers (78%). Seventy-eight per cent of the group had gastrointestinal involvement, and 47% had oesophageal aperistalsis. Left ventricular diastolic dysfunction was observed in 61% of patients, and no patient had experienced a scleroderma renal crisis prior to LT.

All 18 patients were ANA-positive prior to transplantation with a median titre of 1/640. In terms of autoantibody specificity, 44% (n = 8) had ATA I antibodies, 17% (n = 3) had anti-U11/U12-RNP, 11% (n = 2) had anti-RNApol III, 11% (n = 2) had anti-Ro52, and 5% (n = 1) had ACA; no patient had anti-NVL antibodies. No detectable specificity was observed in 22% (n = 4) of patients (Table 1).

### 3.2. Evolution of ANA Status After Lung Transplantation

Following LT, ANA became negative in 7 out of 18 patients (39%) who were categorised as ANA-negative post LT, in a median period of time after LT of 10 (4–29) months. Meanwhile, 11 patients (61%) remained ANA-positive (ANA-positive post-LT group) even when evaluating a longer period of serology status, with a median of 16 (7–38) months. Of the patients in the ANA-positive group, eight patients (44%) exhibited a decrease in ANA titres, and three patients (17%) had stable titres. No patients exhibited an increase in ANA titres during the post-LT follow-up period (Table 1).

A statistically significant difference in pre-LT ANA titres was observed between the two groups. Patients in the ANA-negative post-LT group had significantly lower pre-LT ANA titres compared to those in the ANA-positive group (1/160 vs. 1/640, *p* = 0.004) (Table 2).

Regarding baseline autoantibody specificities: ATA I antibodies were present in 29% of patients in the ANA-negative post-LT group and in 54% (6/11) of patients in the ANA-positive post-LT group. Anti-U11/U12RNP antibodies were found in 43% (3/7) of patients in the ANA-negative post-LT group and in 0% of patients in the ANA-positive post-LT group, with a trend towards statistical significance (*p* = 0.059). Anti-RNApol III antibodies were present in 14% (1/7) of patients in the ANA-negative post-LT group and in 9% (1/11) of patients in the ANA-positive post-LT group. Anti-Ro52 antibodies were detected in 28% (2/7) of the ANA-negative post-LT group and in none of the ANA-positive post-LT group. ACAs were found in only 9% (1/11) of the ANA-positive post-LT group. No statistically significant differences were observed between the two groups in terms of autoantibody specificity (Table 2).

### 3.3. Comparison of Clinical and Epidemiological Characteristics

As shown in Table 2, there were no statistically significant differences in the clinical or epidemiological characteristics of the two groups (ANA-negative post-LT vs. ANA-positive post-LT). Most patients in both groups were female, with a median age of 54 years. The most frequent cutaneous subtypes were dcSSc (3/7, 42% vs. 5/11, 45%) and lcSSc (4/7, 57% vs. 5/11, 45%) in the ANA-negative post-LT and ANA-positive post-LT groups, respectively.

No statistically significant differences were found between the two groups in SSc-related clinical manifestations. ILD was observed in all (7/7) patients in the ANA-negative group and in 81% (9/11) of patients in the ANA-positive group. PH was present in approximately two-thirds of patients in both groups (5/7, 71% vs. 8/11, 73%). A significantly lower pre-transplant FVC was observed in the ANA-negative group, although this may be related to a higher proportion of ILD in this group. No statistically significant differences were observed between the two groups with regard to pre-transplant immunosuppressive therapy. However, a non-statistically significant trend towards a higher cumulative cyclophosphamide dose was noted in the ANA-negative group (Table 2). There were no statistically significant differences in post-LT immunosuppressive treatment between the two groups (Table 3). Almost all patients received a regimen of corticosteroids, mycophenolate mofetil, and tacrolimus after LT. Similarly, early post-LT complications (surgical complications, intensive care unit complications, need for extracorporeal membrane oxygenation after LT, early infectious events, or moderate-to-severe acute LT rejection) did not differ significantly between groups. Interestingly, severe gastroparesis after LT was present in two patients (29%) who became ANA-negative, compared to five cases (45%) in the ANA-positive group (*p* = 0.637). One patient in each group required a temporary percutaneous endoscopic gastrostomy for feeding purposes due to severe gastroparesis. During the follow-up period, there were no differences in late post-LT complications between the two groups either; however, a non-significant trend towards lower prevalence of chronic lung allograft dysfunction (CLAD) was observed in patients who became ANA-negative (29% vs. 54%).

### 3.4. Survival

**Figure 1:**
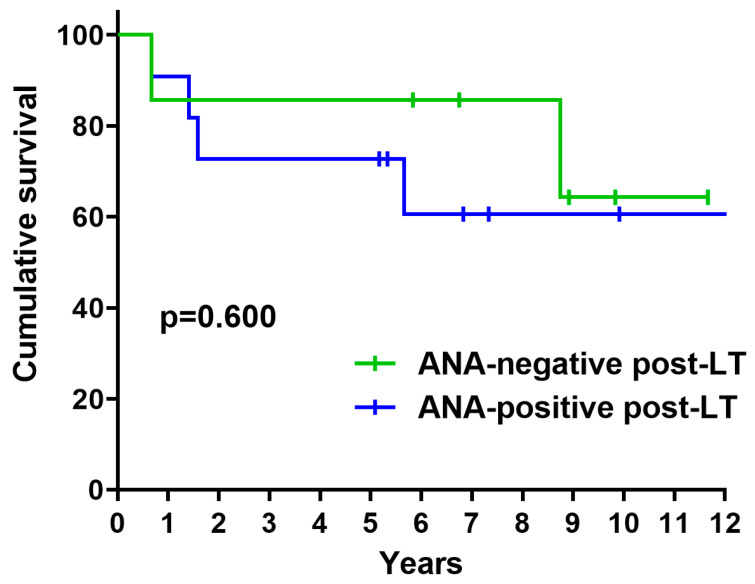
Kaplan–Meier survival curves. Survival comparison between the ANA-negative post-LT group and the ANA-positive post-LT group.

The 1-year and 3-year survival rates in all patients were 89% and 78%, respectively. Seven patients (39%) died during the follow-up period: three from CLAD, two from infection, one from alveolar proteinosis, one from acute LT rejection, and one from malignancy. By August 2025, two patients (28%) had died in the ANA-negative post-LT group and five (45%) in the ANA-positive post-LT group (*p* = 0.637) (Table 3). The median follow-up duration was 105 months for the ANA-negative post-LT group and 68 months for the ANA-positive post-LT group. A non-significant trend towards improved post-LT survival was observed in the Cox proportional hazards analysis. In the unadjusted model, the hazard ratio (HR) was 0.65 (95% CI: 0.11–3.54; *p* = 0.606). After adjustment for age at the time of LT, the HR remained similar (HR 0.58; 95% CI: 0.10–3.30; *p* = 0.537). The cumulative survival rates at 1, 3, and 5 years in the ANA-negative post-LT group were 86% at all time points and 91%, 73% and 73% in the ANA-positive post-LT group, respectively. No statistically significant differences were observed in the Kaplan–Meier survival curves (*p* = 0.600) (Figure 1) (Table 4).

## 4. Discussion

In this study, we analysed the post-lung transplant outcomes of patients with SSc based on changes in their ANA status. No significant clinical differences were observed among patients who became ANA-negative. These patients exhibited lower baseline pre-LT titres, and a trend of higher survival rates was observed compared with patients who remained ANA-positive. Overall, 38.9% of patients became ANA-negative during post-transplant follow-up, 44.4% showed a reduction in ANA titres, and 16.7% maintained stable titres. Notably, none of the patients experienced an increase in ANA levels. Patients who seroconverted had significantly lower ANA titres at baseline. However, no significant differences were found in demographic or clinical features or in post-transplant immunosuppressive regimens. Although the Kaplan–Meier survival curves were not statistically significant, there was a trend towards better cumulative survival in the ANA-negative group (85.7% at both 3 and 5 years) than in the ANA-positive group (72.7% at the same time points).

LT has been a therapeutic option for SSc-related lung disease for over three decades. The first published report dates to 1994, when Levine et al. described nine patients undergoing single-lung transplants, two of whom had SSc [38]. Since then, several studies have evaluated lung transplantation in SSc, although most have included relatively small sample sizes [12,13,39,40]. To date, we have found no studies that focus specifically on post-transplant ANA evolution and its potential prognostic impact. A recent case report described a patient with dcSSc and ILD who underwent bilateral LT, experienced substantial clinical improvement, and showed ANA disappearance during follow-up [24].

The role of SSc-specific autoantibodies in disease pathogenesis remains unclear, although their association with organ involvement and prognosis is well documented. ANAs are recognised as key biomarkers in SSc, often correlating with specific clinical phenotypes and organ manifestations [41]. Some antibodies, such as ATA I and ACA, have been closely linked to disease severity and progression [42,43,44]. While our study found no statistically significant differences in survival based on ANA status post-transplant, the observed trend and the lower baseline ANA titres in the ANA-negative group suggest a possible prognostic value warranting further investigation.

We found no correlation between the immunosuppression therapy administered before or after the LT during follow-up or with early LT complications and ANA clearance. This suggests that ANA disappearance is independent of early LT outcomes. In our cohort, the overall survival rates at 1 and 3 years were 89% and 78%, respectively. The main cause of death was CLAD, followed by infections. These findings are consistent with previous reports. For instance, Crespo et al. evaluated 72 SSc patients who underwent LT and compared them to patients with non-SSc-related ILD. No significant differences were found in 1 and 5-year survival rates (81% and 66%, respectively), although survival in our cohort was slightly higher [39]. Similarly, Fernández-Codina et al. reported no significant survival differences between SSc patients (n = 15) and a large control group of LT recipients without SSc (n = 500), with SSc patients showing 1 and 3-year survival rates of 80% and 65%, respectively. In both studies, infections were the leading cause of death [40]. However, none of these prior studies explored the evolution of ANA status after transplantation.

In our study, survival at 1 and 3 years was 87% in the ANA-negative group, compared to 90% and 73%, respectively, in the ANA-positive group. Although this difference was not statistically significant, it suggests a trend towards better outcomes in ANA-negative patients. Salazar et al. studied over 3200 SSc patients and found that 6.4% were ANA-negative at the time of diagnosis [19]. These patients had a lower incidence of PH and digital ulcers, as well as a higher proportion of men. However, no significant differences in survival were found. The authors proposed that ANA-negative SSc may represent a milder vascular phenotype with a similar overall prognosis.

Some reports have suggested that immunosuppressive therapy may contribute to the disappearance of antibodies. In our cohort, however, analysis of post-LT immunosuppressive treatment did not reveal any clear association with ANA evolution. Indeed, preliminary studies have yielded some evidence that lends support to this hypothesis. For example, Bonroy et al. reported that ANA titres decreased in SSc patients treated with rituximab, and that this was associated with clinical improvement in skin involvement [22]. Similarly, Fürst et al. observed decreases in ANA and ATA I titres and improvements in skin and lung function over a five-year period in patients receiving rituximab [23]. These results suggest a potential connection between B-cell depletion, humoral response, and the clinical manifestation of the disease.

Hinze et al. analysed ILD progression in native versus transplanted lungs in nine SSc patients who underwent single-lung transplantation. They found greater fibrosis progression in the native lung in both imaging (annual change in Hounsfield Units: +2.2 vs. −5.5; *p* = 0.011) and pulmonary volume (annual change: 1.4 mL vs. 27 mL; *p* = 0.03) [45]. This finding suggests that the pathogenic pathways underlying ILD and the autoimmune mechanisms remain active in the native lung and are not fully controlled by immunosuppression. In contrast, in the transplanted lung, autoimmune mechanisms appear to be inactive, and the absence of autoantibodies may account for the more favourable clinical course and improved survival observed in ANA-negative patients in our cohort. We hypothesise that the ANA-negative group had a tendency towards lower CLAD than patients with persistent ANA positivity, which may contribute to CLAD physiopathology and lower survival.

In line with this hypothesis, a recent study by Pinal et al. confirmed that SSc-related autoantibodies can be internalised in the muscle cell nuclei, thus supporting their role in the pathogenesis of inflammatory myopathies associated with scleromyositis [46]. In essence, it appears that autoantibodies may play a pivotal role in perpetuating the feedback loop of immunological autoreactive responses and tissue damage. This mechanism may be shared between systemic sclerosis organ involvement and chronic lung allograft dysfunction (CLAD), where an increased number of non-HLA antibodies has been associated with a higher risk of allograft dysfunction [47].

Further research could involve the longitudinal analysis of other immune biomarkers, such as cytokine signatures and circulating B-cell subsets, to determine whether ANA disappearance correlates with broader immune remodelling post-LT.

This study has some limitations, primarily the small sample size. Larger cohorts and longer follow-up periods are required to validate the potential prognostic significance of ANA disappearance after transplantation. Future research should also focus on the correlation between ANA status and the clinical evolution of SSc manifestations after LT.

Despite these limitations, a key strength of our work is that it is the first study to explore ANA dynamics in a cohort of lung transplant patients with systemic sclerosis and examine their potential association with long-term outcomes.

Multicentre collaborative studies with standardised immunological monitoring are essential in order to address these questions and validate the prognostic relevance of ANA trajectories in larger populations.

## 5. Conclusions

Most patients diagnosed with SSc who undergo LT either become ANA negative or exhibit a progressive decline in antibody titres over time. The only factor associated with higher post-LT ANA disappearance was lower pre-LT ANA titres at baseline. When the two groups were compared, there was a trend towards better survival at three and five years in patients who seroconverted to ANA-negative, with no other significant clinical or epidemiological differences observed. ANA disappearance should be monitored during follow-up and studied as a potential marker of a distinct clinical course. Future multicentre studies should include prospective immunological monitoring to confirm whether ANA loss after LT represents true immunological remission. If confirmed, ANA dynamics could emerge as a valuable surrogate endpoint for immune reprogramming in SSc and other connective tissue diseases requiring transplantation.

## Figures and Tables

**Table 1:** Clinical and epidemiological characteristics of the lung transplanted SSc cohort.

	Total LT-SSc (n = 18)
Female sex	12 (67%)
Age at SSc onset	42.4 (28.0–46.8)
Age at SSc diagnosis	43.2 (30.8–49.2)
Mean age at LT, years	54.4 (±10.9)
Cutaneous subtype	
Diffuse cutaneous SSc	8 (44.4%)
Limited cutaneous SSc	9 (50.0%)
SSc *sine* scleroderma	1 (5.0%)
Bilateral lung transplant	15 (83.3%)
Pulmonary involvement	
ILD	16 (88.8%)
Pulmonary hypertension	13 (72.2%)
FVC% prior to LT	50.7 (36.4–59.8)
DLCO% prior to LT	20 (17.0–33.1)
Raynaud’s phenomenon	17 (94.4%)
Digital ulcers	14 (77.8%)
Telangiectasia	16 (88.8%)
Gastrointestinal involvement	14 (77.8%)
Oesophageal aperistalsis	7 (46.7%)
Gastric	4 (22.2%)
Intestinal	4 (22.2%)
Musculoskeletal involvement	
Arthritis	4 (22.2%)
Myositis	0 (0%)
Tendon contractures	2 (11.1%)
Calcinosis	2(11.1%)
Cardiac involvement	
Pericardial involvement	5 (27.7%)
LV diastolic dysfunction	11 (61.1%)
Systolic dysfunction (LVEF < 50%)	1 (5.0%)
Scleroderma renal crisis	0 (0%)
Late capillaroscopy pattern	5 (27.7%)
Pre-transplant ANA	18 (100%)
Anti-ATA I	8 (44.4%)
Anti-U11/U12RNP	3 (16.7%)
Anti-RNApol III	2 (11.1%)
Anti-Ro52	2 (11.1%)
ACA	1 (5.5%)
Anti-NVL	0 (0%)
ANA-positive post-transplant	11 (61%)
Median ANA titre post-transplant	1/160 (1/160–1/320)
ANA disappearance	7 (39%)
Decrease ANA titres	8 (44.4%)
Stable ANA titres	3 (16.7%)
Increased ANA titres	0 (0%)
Median follow-up, months	29.0 (8.0–62.5)
Accumulative survival global n (%)	13 (72.2%)

Continuous variables are presented as median (interquartile range); categorical variables as number (percentage). LT: lung transplantation; SSc: systemic sclerosis; ILD: interstitial lung disease; LV: left ventricle; LVEF: left ventricular ejection fraction; ANA: antinuclear antibodies; Anti-ATA I: anti-topoisomerase antibody; Anti-RNApol III: anti-RNA polymerase III antibody.

**Table 2:** Clinical, epidemiological, and immunological characteristics by post-LT ANA status.

	ANA-Negative Post-LT (n = 7)	ANA-Positive Post-LT (n = 11)	*p*-Value
Female sex	5 (71.4%)	7 (63.6%)	1.000
Age at SSc onset	42.2 (34.7–46.8)	42.7 (27.9–46.8)	0.928
Age at SSc diagnosis	42.2 (36.4–49.9)	43.7 (30.1–49.0)	0.892
Median age at LT, years	54.2 (46.1–56.4)	54.7 (47.8–59.4)	0.526
Cutaneous subtype			
Diffuse cutaneous SSc	3 (42.9%)	5 (45.5%)	
Limited cutaneous SSc	4 (57.1%)	5 (45.5%)	1.000
SSc *sine* scleroderma	0 (0%)	1 (9.1%)	
Bilateral transplant	6 (85.7%)	9 (81.8)	1.000
Pulmonary involvement			
ILD	7 (100%)	9 (81.85)	
PH	5 (71.4%)	8 (72.7%)	0.497
PH group 1	2 (28.6%)	6 (54.5%)	0.931
PH group 3	3 (42.9%)	2 (18.2%)	
FVC% prior to LT	36.3 (30.9–52.0)	54.7 (44.7–73.7)	0.033
DLCO% prior to LT	19.0 (15.7–19.8)	30.4 (17.6–39.0)	0.111
Raynaud’s phenomenon	6 (85.7%)	11 (100%)	0.389
Digital ulcers	6 (85.7%)	8 (72.7%)	1.000
Telangiectasia	6 (85.7%)	10 (90.0%)	1.000
Gastrointestinal involvement	6 (85.7%)	8 (72.7%)	0.480
Oesophageal	6 (85.7%)	7 (63.6%)	0.316
Aperistalsis	3 (42.9%)	4 (50%)	1.000
Ineffective peristalsis	4 (57.1%)	3 (37.5%)	1.000
Normal	0 (0%)	1 (12.5%)	1.000
Gastric	3 (42.9%)	1 (9.1%)	0.240
Gastroparesis	4 (57.1%)	2 (18.2%)	0.141
Intestinal	2 (28.6%)	2 (18.2%)	0.764
Musculoskeletal involvement			
Arthritis	0 (0%)	4 (16.0%)	0.119
Myositis	0 (0%)	0 (0%)	1.000
Tendon contractures	1 (14.3%)	1 (9.1%)	1.000
Calcinosis	1 (14.3%)	1 (9.1%)	0.393
Cardiac involvement			
Pericardial involvement	1 (14.3%)	4 (36.0%)	0.596
LV diastolic dysfunction	4 (57.1%)	7 (63.6%)	0.871
LVEF < 50%	0 (0%)	1 (9.1%)	1.000
Scleroderma renal crisis	0 (0%)	0 (0%)	1.000
Late capillaroscopy pattern	2 (28.6%)	3 (27.3%)	0.934
Baseline ANA	7 (100%)	11 (100%)	1.000
ATA I	2 (28.6%)	6 (54.5%)	0.367
Anti-U11/U12RNP	3 (42.9%)	0 (0%)	0.059
Anti-RNApol III	1 (14.3%)	1 (9.1%)	1.000
Anti-Ro52	2 (28.6%)	0 (0%)	0.367
ACA	0 (0%)	1 (9.1%)	0.137
Immunosuppression prior to LT	7 (100%)	10 (90.9%)	1.000
Corticosteroids	6 (85.7%)	8 (72.7%)	1.000
Median prednisone dose (mg/d)	5.0 (4.4–11.2)	5 (5.0–7.5)	0.890
Mycophenolate acid	7 (100%)	7 (63.6%)	0.119
Median mycophenolate dose (mg/d)	720 (720–1440)	1440 (720–2160)	0.258
Cyclophosphamide	6 (85.7%)	6 (54.5%)	0.316
Median accumulated cyclophosphamide dosage (g)	12.2 (3.3–13.4)	4.0 (1.2–10.1)	0.296
Rituximab	4 (57.1%)	4 (36.4%)	0.630
Azathioprine	3 (42.9%)	4 (36.4%)	1.000
Median ANA titres at baseline (IQR)	1/160 (1/160–1/320)	1/640 (1/480–1/960)	0.004

Continuous variables are presented as median (interquartile range); categorical variables as number (percentage). ANA: antinuclear antibodies; LT: lung transplantation; SSc: systemic sclerosis; ILD: interstitial lung disease; PH: pulmonary hypertension; FVC%: forced vital capacity percentage; DLCO%: carbon monoxide diffusion capacity percentage; LV: left ventricle; LVEF: left ventricular ejection fraction; ACA: anti-centromere antibody; ATA I: anti-topoisomerase antibody; Anti-RNApol III: Anti-RNA polymerase III antibody; mg/d: milligrams per day; g: accumulated grams.

**Table 3:** LT immunosuppression therapy, LT complication, and survival characteristics by post-LT ANA status.

	ANA-Negative Post-LT (n = 7)	ANA-Positive Post-LT (n = 11)	*p*-Value
Immunosuppression therapy			
Corticosteroids	7 (100%)	11 (100%)	1.000
Mycophenolate mofetil	7 (100%)	6 (54.5%)	0.101
Tacrolimus	7 (100%)	11 (100%)	1.000
Rapamycin	0 (0%)	4 (36.0%)	0.119
Early LT complications			
Surgical complications	4 (57.1%)	6 (54.5%)	1.000
ICU complications	4 (57.1%)	6 (54.5%)	1.000
ECMO	2 (28.6%)	1 (9.1%)	0.528
Gastroparesis	2 (28.6%)	5 (45.5%)	0.637
Respiratory complications	2 (28.6%)	3 (27.3%)	1.000
Early infections	2 (28.6%)	5 (45.5%)	0.637
Moderate-severe acute LT rejection	1 (14.3%)	5 (45.5%)	0.316
Late LT complications			
CLAD	2 (28.6%)	6 (54.5%)	0.367
Outcome			
Median follow-up, months	105 (70–118)	68 (19–119)	0.526
Mortality	2 (28.6%)	5 (45.5%)	0.637
Cumulative survival			
1-year survival	6 (85.7%)	10 (90.9%)	1.000
3-year survival	6 (85.7%)	8 (72.7%)	0.316
5-year survival	6 (85.7%)	8 (72.7%)	0.316

Categorical variables are presented as numbers (percentages). ANA: antinuclear antibodies; LT: lung transplantation; ECMO: extracorporeal membrane oxygenation; CLAD: chronic lung allograft dysfunction.

**Table 4:** Subjects at risk.

	Baseline	1 y	2 y	3 y	4 y	5 y	6 y	7 y	8 y	9 y	10 y	11 y	12 y
ANA-positive post-LT	11	10	8	8	8	8	5	4	3	3	2	2	2
ANA-negative post-LT	7	6	6	6	6	6	5	4	4	2	1	1	0

## Data Availability

The original contributions presented in the study are included in the article, further inquiries can be directed to the corresponding author.
